# Further insights into the operation of the Chinese number system: Competing effects of Arabic and Mandarin number formats

**DOI:** 10.3758/s13421-020-01065-x

**Published:** 2020-07-09

**Authors:** Philip T. Quinlan, Dale J. Cohen, Xingyu Liu

**Affiliations:** 1 Department of Psychology, The University of York, Heslington, York, Y010 5DD UK; 2 Department of Psychology, The University of North Carolina at Wilmington, Wilmington, NC USA; 3 Laboratory of Applied Experimental Psychology, Beijing Normal University, Beijing, People’s Republic of China

**Keywords:** Mathematical cognition, Number processing, Chinese number system

## Abstract

Here we report the results of a speeded relative quantity task with Chinese participants. On each trial a single numeral (the probe) was presented and the instructions were to respond as to whether it signified a quantity less than or greater than five (the standard). In separate blocks of trials, the numerals were presented either in Mandarin or in Arabic number formats. In addition to the standard influence of numerical distance, a significant predictor of performance was the degree of physical similarity between the probe and the standard as depicted in Mandarin. Additionally, competing effects of physical similarity, defined in terms of the Arabic number format, were also found. Critically the size of these different effects of physical similarity varied systematically across individuals such that larger effects of one compensated for smaller effects of the other. It is argued that the data favor accounts of processing that assume that different number formats access different format-specific representations of quantities. Moreover, for Chinese participants the default is to translate numerals into a Mandarin format prior to accessing quantity information. The efficacy of this translation process is itself influenced by a competing tendency to carry out a translation into Arabic format.

## Introduction

Numerical quantities can be expressed in different formats. For example, the quantity four can be expressed as the Arabic digit (e.g., “4”), the written word (“four”), and the spoken word (/fôr/). Early attempts to understand how the human number system operates were based on positing separate encoding modules for the corresponding input formats together with a single abstract code used to access stored quantity information (e.g., see the *abstract code model*: McCloskey, 1992; Sokol, McCloskey, Cohen, & Aliminosa, 1991; and the *triple code model*: Dehaene, 1992). A basic idea is that, at an early stage of processing, numbers conveyed in terms of different input formats are converted to a common abstract code. According to such accounts, once number processing proceeds in terms of this abstract code, subsequent operations are assumed to be functionally independent of the visual format of the input stimulus.

Fairly rapidly, however, evidence emerged that was used to question this generally accepted framework for thinking. Some of this evidence came from the study of Chinese-English bilinguals (Campbell & Epp, 2004; Campbell, Kanz, & Xue, 1999). Such participants were selected because they are acquainted with two different number notation systems (i.e., Arabic and Mandarin, respectively). For these participants, different kinds of numerical symbols are naturally associated with the same quantities, for instance both “四” and “4” signify the quantity four. Campbell and colleagues therefore tested these bilinguals in a range of number-processing tasks. One such task was the relative quantity task. On each trial, two numerals in either Arabic or Mandarin number formats were presented and, under reaction time (RT) instructions, participants had to respond to which was smaller. The data revealed that participants responded faster as the numerical distance between the two numbers increased – this has come to be known as the *numerical distance effect*. Importantly, the numerical distance effect was much larger when Mandarin numbers were presented than when Arabic numbers were. Campbell and colleagues explained the data by positing an *encoding-complex model* of number processing. In this model, the Mandarin numerical symbols map directly onto a knowledge store that contains Mandarin number facts: A separate knowledge store is posited for Arabic number facts. The Arabic symbols map onto both stores but the Mandarin symbols only map directly onto the Mandarin store. Such differential access was used to account for the different patterns of performance that the bilinguals produced across a battery of number processing tasks. Moreover, the conclusion was that performance in such tasks reflects, not so much operations taking place in a common abstract code, but interactive operations carried out in format-specific modules and a common number system.

A quite different appraisal of these issues has more recently been reported by Cohen, Warren, and Blanc-Goldhammer (2013). They discussed an alternative but very similar architecture, labeled the *Multiple Representation Model*. They considered the mapping of different input forms of numbers – printed English number words, spoken English number words and Arabic digits – onto format-specific quantity representations. They posited different encoding modules for the different formats each of which provides direct access to its own set of quantity representations (see also Cohen Kadosh & Walsh, 2009, and Blankenberger & Vorberg, 1997, who considered numerical operations entrained by the presentation of number words, digits, and dice faces). Critically however, in the Multiple Representation model, crosstalk between the different encoding modules was posited, but the separate quantity representations were assumed to be insulated from one another.

Cohen et al. (2013) explored the nature of these different stimulus encoding processes as a precursor to demonstrating how such processes influence performance in certain numerical tasks. To appreciate this, however, it is important to acknowledge the prior work of Cohen (2009). Previously Cohen (2009) had generated measures of physical similarity between the various Arabic digits and had used a task in which participants were timed to respond as to whether a presented visual digit (the probe) was a “5” (the standard). Associated quantities are irrelevant in this task because participants are only required to identify the numerals. RTs were analyzed both as a function of the numerical distance between the probe and the standard, and as a function of their physical similarity. A key prediction was that performance would reflect how readily the physical structure of the probe and standard are confused. For instance, RTs ought to be shorter when the probe is less physically similar to the standard (e.g., “1” vs. “5”) than when it is more physically similar to the standard (e.g., “6” vs. “5”). The results of the experiment were clear: RTs were better predicted by physical similarity of the numerals than by numerical distance between their associated quantities.

An important aspect of the task described by Cohen (2009) is that it involved speeded digit identification that logically could be completed without having to access stored quantities. Of some further interest, therefore, is the question of the degree to which the physical similarity of digits might influence performance when the task does depend on accessing stored quantities. In this respect, the work of Cohen et al. (2013) is germane because one of the tasks they used was a speeded relative quantity task. For example, on each trial a digit and word would be visually presented (in Experiment 2), one above the other, and the participant was timed to respond as to whether the bottom stimulus was numerically larger or smaller than the top stimulus. Perhaps, unsurprisingly, numerical distance between the presented probes was found to be a strong predictor of performance, but, in addition, the degree of physical similarity between the stimuli also emerged strongly as a corresponding predictor. Such a pattern of results clearly emphasizes the fact that the physical characteristics of the numerical stimuli cannot be ignored in accounting for performance across a range of numerical tasks. Indeed, such a conclusion has now extensive support in the literature (see Cohen, 2009, 2010; Cohen & Quinlan, 2016; Defever, Sasanguie, Vandewaetere, & Reynvoet, 2012; Garciá-Orza, Perea, Mallouh, & Carreiras, 2012; Lin & Göbel, 2019; Wong & Szücs, 2013; Zhang, Xin, Feng, Chen, & Szücs, 2018).

Further tests of these ideas are possible if we adopt the rationale spelt out by Cohen et al. (2013). They addressed the issue of how numerical comparisons are carried out if the numbers, to be compared, are presented in different formats. The abstract code model is clear in predicting no effects of physical similarity in a relative quantity task because quantities are represented in a common abstract code. Quantities can only be accessed once translation to this code has been completed and then they can be directly compared. The Multiple Representation model, however, is more nuanced than this because the different number formats are each associated with a different format-specific representation of stored quantities and so this, in a sense, forces the problem backwards. Direct comparisons of quantities cannot be carried out on different surface formats nor in their corresponding different abstract formats. As a consequence, the Multiple Representation model proposes that such comparisons can only be carried out if the different surface formats are converted into a common underlying format so as to access the same quantity information. For instance, if “four” is to be compared to “5” then, during encoding, “four” is translated into “4” so that both number formats access the same quantity information; in this case that associated with Arabic digits. If such translation processes do operate then effects relating to the physical similarity of Arabic digits should emerge even when some of number information is conveyed in words. Critically such effects were present in the data reported by Cohen et al. (2013) in their cross-format comparison tasks, in line with the predictions of the Multiple Representation Model.

It remains possible that converging evidence for these ideas can be garnered from the study of Chinese participants. Chinese participants are acquainted with both Arabic and Mandarin number formats. In Chinese culture, whereas Arabic numbers are used in the main for number tasks – arithmetic and other mental calculations – Mandarin numbers are not used in this way and function as number words and in expressions of time such as weekdays and months (Campbell & Epp, 2004, p. 231). According to the Multiple Representation Model therefore, it can be assumed that there are separate encoding modules for these different number formats and different quantities associated with the different surface formats also exist.

In pursuing these ideas, we studied a timed, relative-quantity task with Chinese participants. On each trial in the experiment a single numeral was presented centrally on a computer screen and participants were tasked with responding as quickly and as accurately as to whether its associated quantity was numerically greater than or less than five. The experiment was divided into two parts such that within each part only Arabic or Mandarin numbers were presented.

The Multiple Representation model makes very particular predictions as to what performance in these tasks will reveal. Primarily the hypothesis is that that effects of physical similarity of the surface format of the numbers will be revealed. However, there are two formats that need to be considered and it remains to be seen whether performance will reflect the influence of encoding in terms of the Arabic number system or the Mandarin number system. Perhaps the most straightforward predictions are as follows. When Arabic numbers are used then effects of physical similarity will reflect similarity computed over the Arabic surface format of the digits. In contrast, when Mandarin characters are used then effects of physical similarity will reflect similarity computed over the Mandarin surface format. Such predictions are based on assuming that the different number formats engage different forms of encoding and that each format is associated with its own format-specific stored quantities. If, however, both Arabic and Mandarin formats elicit physical similarity effects of the same format (e.g., Mandarin), then one can conclude that the Chinese participants converted the numerical symbols into a single format (e.g., Mandarin) to complete the quantity comparison task.

It is also important to be clear at the outset that the experimental paradigm is a speeded quantity estimation task – Is the presented digit greater than or less than five? Given this, we would expect to find a numerical effect in the data. However our primary interest was in examining the degree to which metrics of physical similarity also affect performance. We take it that the current task provides a window on early perceptual encoding processes and the accessing of stored quantity information from visual input (see Cohen & Quinlan, 2019, for a detailed exposition of the kinds of processes that are of interest here). In this respect, the data cannot provide any further insights into how arithmetic operations may be influenced by the surface (i.e., visual) format of the numbers and this contrasts with the other cited work in which format effects have been explored in the context of mental arithmetic (see Blankenberger & Vorberg, 1997; Campbell & Epp, 2004; Campbell et al., 1999).

## Method

### Measures of physical similarity for digits

Cohen (2009) set out a method for calculating a physical similarity function for the Arabic digits. Each digit was formed from a digital figure 8 (i.e., “⊟” as used in digital clocks) that itself comprised seven-line segments. “P,” the physical similarity between any two digits, was defined as:

1 $$ \mathrm{P}=\frac{O}{D} $$

where O is the number of line segments that the two digits share (the overlapping segments) and D is the number of the remaining non-overlapping line segments. We will refer to the physical similarity measures for the Arabic digits as PSA.

**Fig. 1:**
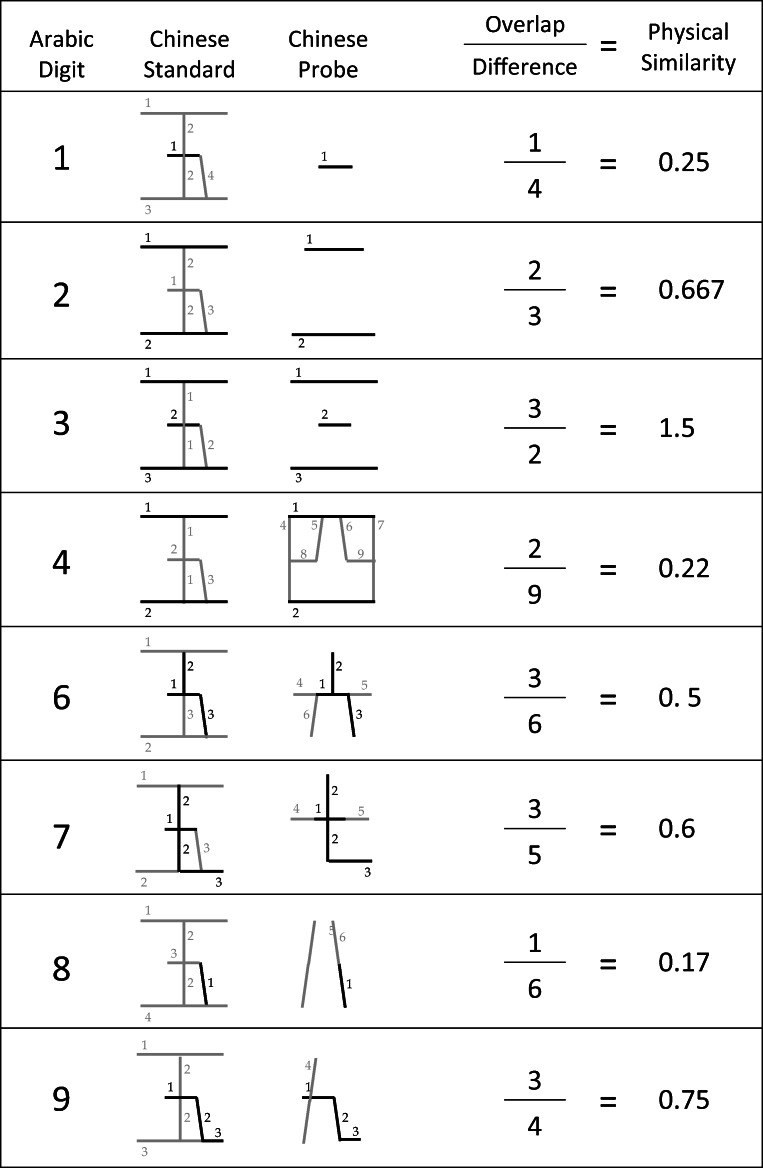
The calculation of the physical similarity function between each Mandarin numeral and the target “五”

Here we used exactly the same method to compute physical similarity measures for Mandarin digits (henceforth, PSM). The Mandarin numbers were rendered into line segments and the computations of physical similarity are as shown in Fig. 1.

### Measures of the numerical distance for digits

To assess the influence of numerical distance, we calculated the Welford function. Moyer and Landauer (1967) showed that the Welford function models the numerical distance effect for a set of integers (e.g., 0 –9 ):

2 $$ \mathrm{RT}=a+\kern0.5em k\ast \log \left[L/\left(L\ S\right)\right], $$

where *a* and *k* are constants, *L* is the larger quantity, and *S* is the smaller quantity (Welford, 1960). The Welford function is particularly well suited to model numerical distance because it also models the general finding that larger quantities (e.g., 8 vs. 9) are more difficult to distinguish than smaller quantities (e.g., 1 vs. 2; often referred to as the *size effect*; Krajcsi, Lengyei, & Kojouharova, 2016).

### Participants

In total 78 undergraduates at the Beijing Normal University were tested in the experiment; however, data for 14 participants failed to save properly. Across the final 64 participants, the average age was 21 years, 1 month, and 27 were male.

### Design and procedure

On each trial in the experiment the participant had to respond as quickly and accurately as possible as to whether the displayed number was greater than or less than the number five. The designated keyboard response keys were “D” and “K.” In the experiment proper, performance with Arabic numbers and Mandarin numbers was tested in separate runs of blocks of trials, namely, the *Arabic condition* and *the Mandarin condition*, respectively. In each case, there were eight practice trials followed by four blocks of 120 experimental trials. Each of the numbers 1–4 and 6–9 were sampled equally often within each of the blocks of trials with the presentation of the particular numbers being determined on a random basis.

The 64 participants were randomly divided equally into four counterbalancing groups in which the order of testing Arabic versus Mandarin numbers was taken into account and within this, half the participants responded “greater than five” with a “D” key press and half responded “greater than five” with a “K.”

The experiment was programmed in JavaScript in the context of the jsPsych library (de Leeuw, 2015). This meant that the experimental script was disseminated as a web link and participants were allowed to complete the experiment using which ever computer was at their disposal whenever they chose to engage with the task. The script checked to make sure that the Microsoft Yahei font was installed on the machine. Participants were instructed to make sure that the only software to be currently active was a web browser and that all other forms of alerts should be switched off. They were also instructed to complete the experiment in a quiet place away from any distraction.

A single number was presented on a trial and remained until either a “D” or “K” keyboard press was registered or a response deadline of 1,500 ms expired. Following this there was a blank period 500 ms. On correct trials there was a further 600-ms blank interval whereas on incorrect trials “xxx” was presented for 300 ms followed by a 300-ms blank interval.

All the text in the experiment was presented in white on a black screen. The numbers were cast into Arial Microsoft Unicode font size 2.5 em. The experiment was run under Full Screen mode in which the screen was rendered black.

## Results

### Data analysis

We analyzed the data in a similar fashion to that reported by Cohen et al. (2013). By adopting the analysis procedure of Cohen et al. (2013), we strongly limit the potential for ad hoc statistical bias (Simmons, Nelson, & Simonsohn, 2011). Central to this approach is mixed effects regression (Faraway, 2006) that takes account of both within- and between-participant variance. We are aware that this is different from more traditional reporting that relies on ANOVA, but, subsequently, we focus on inter-individual differences in the tasks, and repeated-measures ANOVA “does not distinguish variance dues to systematic interindividual differences from error variances” (Van Dongen, Olofsen, Dinges, & Maislin, 2004, p. 149).

To assess the influence of physical similarity and numerical distance, we calculated three predictor variables: two measures of physical similarity (i.e., PSM, and PSA as described by Cohen 2009) and the Welford function. The two measures of physical similarity provide alternative predictions for these RT on the assumption that performance is affected by how similar the probe digit is to “5” and “五,” respectively. Table 1 provides the corresponding correlation matrix showing the overall pairwise correlation coefficients for the three predictor variables computed over the eight integers 1–4, 6–9. Given the small number of integers (i.e., 8 integers with a *df* = 6), however, none of the coefficients were statistically significant, all *p*s > .05, two tailed tests. Nevertheless, because these variables are objective measures of the hypothesized constructs (rather than samples), statistical inference is not particularly relevant here. Effect size is a more informative measure. PSM correlates only weakly with the other two variables (thus a small effect size), whereas PSA and the Welford function are strongly correlated and thus have a large effect size (Cohen, 1992).^1^

**Table 1:** Correlation matrix for the three predictor variables: Physical Similarity of Arabic Numerals (PSA), Physical Similarity of Mandarin Numerals (PSM), and the Welford Function that captures the numerical distance effect

	PSA	PSM
PSM	0.08	
Welford	0.62	-0.19

Prior to analysis, we standardized each predictor variable to determine its relative influence on RT. We then used the standardized variables as predictors in a simultaneous mixed model regression with participant as a random variable. Each of the predictor variables provides a particular estimate of RT for each target integer and the regression allows us to establish the goodness of fit between these estimates and performance. Our primary interest is in the degree to which each predictor variable accounts for a statistically reliable amount of the overall variance.

As further clarification, we used mixed effects regression to fit functions to each individual’s raw data and because of this the resulting summary statistics can appear to be trivially small. In commenting upon this, Cohen et al. (2013) noted that in alternative analyses that rely on aggregating over individual trials and participants, *r*^*2*^ values can be of the order of 0.8, whereas analyses that take into account individual trial variance typically result in corresponding *r*^*2*^ of 0.2. However, Cohen et al. (2013) reported that whereas their mixed regression produced *r*^*2*^ values of ~.2 these scaled up accordingly when the data were averaged over trials and participants. They also noted that *r*^*2*^ values of the order of .2 correspond to effects of medium to large size (p. 364, after Cohen, 1992). It is also useful to be aware that because the tests are based on consideration of individual participant responses the corresponding *df*s tend to be noticeably larger than what might have otherwise been expected.

Prior to analysis, we normalized the RT data by taking its (natural) log transform. We did also carry out the same analyses on the untransformed data, and the same general patterns obtained. Nonetheless, we feel it is more appropriate to report the results with the transformed data because of the assumptions that underpin the statistical tests. Finally, to eliminate outliers, we removed the fastest and slowest .5% of the data and we also removed one participant’s data from the Arabic condition because the participant likely reversed the response keys (error rate > 0.96; likely a reversal of keys).^2^

### Overall indices of performance

Initially we simply compared overall performance as reflected in mean correct RTs: paired *t*-tests determined that responses in the Arabic condition were reliably faster, (log(RT): M = 6.16, i.e., 473 ms, SD = 0.12), than were those in the Mandarin condition, (log(RT): M = 6.19, i.e., 488 ms, SD = 0.13), *t*(62) = 4.16, *p* < .001, *d* = 0.52. In addition, responses in the Arabic condition were reliably more accurate (error rate: M = .036, SD = .026) than those in the Mandarin condition (error rate: M = .04, SD = .025), *t*(62) = 2.15, p = .04, *d* = 0.27. There is no evidence therefore of any speed/accuracy tradeoff in the data.

### Performance broken down by condition

**Fig. 2:**
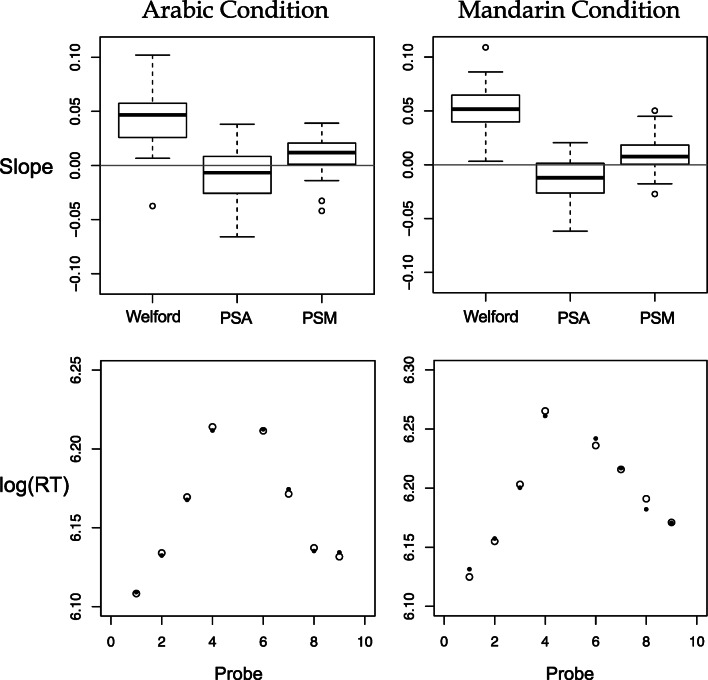
Summaries of the model fits. The top row shows boxplots displaying the distribution of slopes (i.e., the β values associated with each predictor) for participant for each predictor variable in the Arabic and Mandarin conditions, respectively, left and right figures. The bottom row shows a summary of the fits of the model. Specifically, the white circles are the reaction time (RT) data collapsed over participant, and the black circles are the fit data from the model, collapsed over participant in the Arabic and Mandarin conditions, respectively left and right figures

Next, we carried out separate regression analysis for the Arabic and Mandarin condition data, respectively. Figure 2, top row, provides summary data of the various predictors in graphical form. Specifically the data plotted are box and whisker plots of the individual βs (i.e., the slopes) computed on a participant-by-participant basis for the three predictors, respectively. Because each regression tested the statistical reliability of three slopes, we applied a Bonferroni correction requiring an alpha of .017. Figure 2, bottom row, shows a summary of the actual data and fit for the data collapsed over participants. The white circles are the actual RT data and the black circles are the fits to these data.

In the Arabic condition, there was a statistically significant effect of the Welford function (slope = 0.04), *F*(1, 438) = 305.51, *p* < .0001, and a statistically significant effect of the PSM, (slope = 0.01), *F*(1, 438) = 26.67, *p* < .0001. There was also a marginal effect of the PSA, but in the opposite direction to that previously reported (slope = -0.01), *F*(1,438) = 5.94, *p* < .015 (overall model fit, *r*^*2*^ = .3).

In the Mandarin condition, there was a statistically significant effect of the Welford function (slope = 0.05), *F*(1, 435) = 364, *p* < .0001, and a statistically significant effect of PSM, (slope = 0.01), *F*(1, 435) = 20.59, *p* < .0001. There was also a statistically significant effect of PSA, but in the opposite direction than predicted (slope = -0.014), *F*(1, 435) = 16.23, *p* < .0001, (overall model fit, *r*^*2*^ = .31).

### Inter-participant effects

**Fig. 3:**
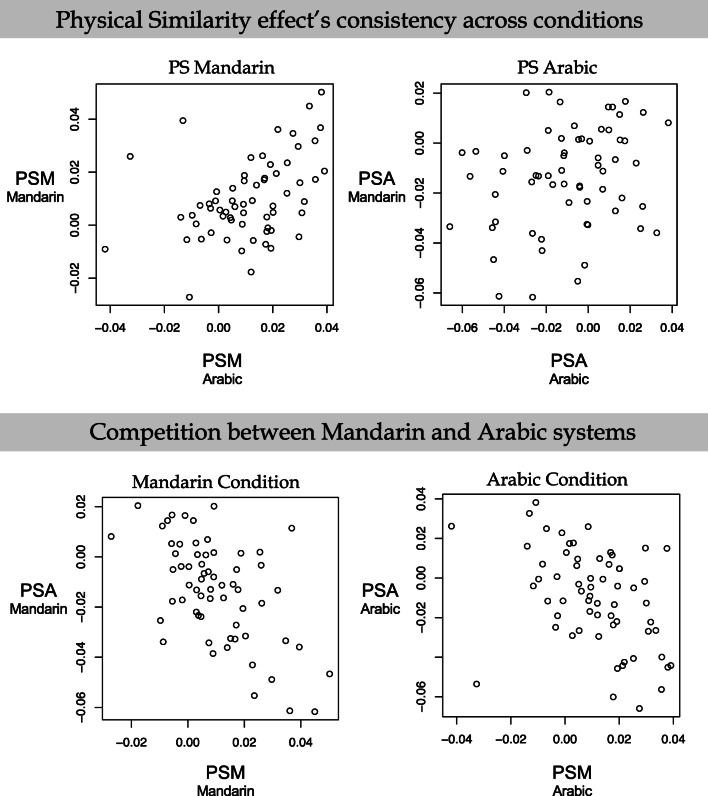
Scatter plots displaying the relation between the physical similarity measures for the Arabic digits (PSA) and physical similarity measures for Mandarin digits (PSM) slopes. The top left-hand panel shows a scatter plot of how the participant slope values for PSM varied across the Mandarin and Arabic conditions – the positive correlation reflects the fact that the influence of PSM was the same both conditions. The top right-hand panel shows a scatter plot of how the participant slope values for PSA varied across the Mandarin and Arabic conditions – the scatterplot reveals the influence of PSA in the Mandarin condition was unrelated to that in the Arabic conditions. The bottom left-hand panel shows a scatter plot of how the participant slope values for PSM and PSA varied within the Mandarin condition. The bottom right-hand panel shows a scatter plot of how the participant slope values for PSM and PSA varied within the Arabic conditions

It is possible to ascertain a better understanding of how performance is influenced by knowledge of the two different number formats by carrying out analyses broken down by participant. Initially we report how the influence of PSM and PSA (i.e., their corresponding β values) varied across the Mandarin and Arabic conditions, respectively (see Fig. 3, top panel). That is, we examined how the influence of PSM expressed itself across the Mandarin and Arabic conditions, (Fig. 3 top left panel) and, similarly, we examined how the influence of PSA expressed itself across the Mandarin and Arabic conditions (Fig. 3 top right panel). An overall positive correlation across participants would reveal that the influence of the particular format (either Mandarin or Arabic) was the same in both the Mandarin and Arabic conditions. Next, we examined how within each condition the different formats exert their influence. Here we carried out separate analyses for the Mandarin (Fig. 3 bottom left panel) and Arabic condition (Fig. 3 bottom right panel). An overall positive correlation across participants in either case would reveal that the influence of both of the formats was the same in the Mandarin and Arabic conditions. Because we calculated four correlations, we applied a Bonferroni correction, requiring an alpha of .0125.

The analyses revealed that there was a statistically reliable positive correlation between the slopes for PSM across the Mandarin and Arabic conditions, *r* = .42, *t*(61) = 3.6, *p* < .001, (see Fig. 3, top left-hand panel). However, the correlation between the slopes for PSA across the Mandarin and Arabic conditions failed to reach statistical significance, *r* = .26, *t*(61) = 2.1, *ns* (see Fig. 3, top-right hand panel). In addition, there were statistically significant negative correlations between the slopes for PSM and PSA in the Mandarin condition, *r* = -.58, (see Fig. 3, bottom left-hand panel) *t*(61) = -5.5, *p* < .001, and in the Arabic condition, *r* = -.43, *t*(61) = -3.7, *p* < .001 (see Fig. 3, bottom right-hand panel).

## General discussion

In many respects the data are clear in showing how the surface format of the numerals critically influences judgments about their associated quantities. The data add to the growing body of evidence that endorses this: such effects have been shown with Arabic numerals (see e.g., Cohen, 2019), Persian/Indian numerals (Garciá-Orza et al., 2012) and now Mandarin numerals. Here we chose to examine performance in a relative quantity task in which a single digit was presented on a trial (the probe) and the participant under RT instructions judged whether the digit was greater than or less than five (the standard). We expected there to be evidence of the numerical distance effects in the data (i.e., as evidenced by statistically reliable fits with the Welford function) and there are, but in addition we wanted to examine the degree to which knowledge of different number formats would also influence performance. To this end, we tested native Chinese participants with both Arabic and Mandarin digits and, in both cases, performance was modulated by the how physically similar the presented digit was to the Chinese character “五” (i.e., five).

Previously Cohen (2009) showed that when American participants were tested with Arabic digits (i.e., as in the Arabic condition) their responses were influenced by how physically similar the presented digit was to “5.” The more similar the probe was to the standard the slower participants were to respond. Detailed simulations of performance in this task are provided by Cohen and Quinlan (2016). By analogy, we found a similar pattern of performance when Chinese participants were presented with probes presented in Mandarin. That is, the speed of responding was influenced by how physically similar the presented probe was to the standard “五.” To show this, we first computed new indices of physical similarity (i.e., PSM) using the same method as Cohen (2009). We then used these indices as predictors in mixed effects regression models. These core findings extend the evidence-base that performance in simple number tasks cannot be fully accounted for by examining quantity information alone. Both the physical format of the numbers and knowledge of different formats can also play significant roles.

Aside from bolstering and extending existing evidence, the present data have produced some intriguing new findings. The data revealed that the PSM indices accounted for significant variance in both the Chinese and the Arabic conditions. The fact that PSM predicted performance in the Arabic condition indicates that, even though the probes were presented in Arabic, responses were influenced by how the corresponding Mandarin probe was similar to the Mandarin standard. The Multiple Representation model (Cohen et al., 2013) provides a ready explanation of this. Specifically, such a pattern suggests that when an Arabic probe is presented, a process of translation occurs whereby the Arabic digit is converted into a Mandarin format and via this format quantity information is accessed and compared.

To better understand this process, we examined how performance varied over individuals. The data revealed informative correlations between the PSM and PSA parameters across conditions and the size of the influence of PSM correlated positively across Mandarin and Arabic conditions (Fig. 3 top-left panel). This demonstrates that the influence of the Mandarin notation was stable within individuals when they carried out the relative quantity task regardless of the format of the probe. This stability is striking when compared to the instability of the Arabic notation’s influence. Specifically, the size of the influence of PSA did not correlate across Chinese and Arabic conditions (Fig. 3 top-right panel).

The instability of the PSA across conditions suggests that Arabic notation is not processed as automatically nor as regularly as Mandarin notion. This conclusion is further evidenced by (a) the subsidiary analyses broken down by the order of testing of the conditions (see Appendix) that reveals the influence of PSA is *only* present when the Arabic condition is presented first, and (b) the finding that the slope parameter for PSA is opposite in direction to those previously reported in western populations (see, e.g., Cohen et al., 2013). Generally, increased similarity of a probe to the standard slows RT. In the current data for the PSA parameter, however, increased similarity of a probe to the standard shortened RT.

We believe the opposite parameter for PSA and the instability of PSA provide information about the encoding/translation processes that precede accessing quantity information (see Cohen & Quinlan, 2016). These findings are best understood in conjunction with the correlation between PSA and PSM. Specifically, there is a negative correlation between the PSM and the PSA effect across individuals in the Mandarin (Fig. 3 bottom-left panel) and Arabic condition (Fig. 3 bottom-right panel). Thus, individuals who show positive slopes for PSM tend to show negative slopes for PSA and this relation varies systematically across individuals. This suggests that Arabic and Mandarin processes are carried out in tandem but in a mutually competitive way and that the control/scheduling of these processes varies systematically across individuals.

The idea that processes associated with different number formats operate in mutually interactive fashion has also been discussed by Campbell and colleagues in their encoding-complex account (see, e.g., Campbell & Epp, 2004). Whereas in that account a single store of abstract quantities is posited that is accessible from all formats, in the Multiple Representation model of Cohen et al. (2013) each surface format has its own associated stored quantities. To compare quantities represented by different surface formats implies translating the digits into a common representation from which the same format-specific quantities can be transformed. The present data show that different individuals vary in the degree to which particular format-specific processes are weighted. The more the Mandarin system dominates, the less the influence of the Arabic system and vice versa.

This is the first time that competition between number formats has been demonstrated, whereby activation of one format inhibits another format. Such a competition may well increase efficiency if both formats can potentially activate quantity information, but their quantity codes cannot be directly compared. The inability to directly compare the quantity information relegates one of the formats as superfluous. As such, that format simply adds noise to the system. The inhibition of that format will therefore reduce the noise in the system, thus aiding the comparison process.

Our current behavioral data may also help inform interpretation of neuroimaging data on number processing. Tang et al. (2006) conducted an fMRI experiment on number processing that compared Chinese participants with non-Chinese English-speaking participants across a range of visual judgements. In one critical task, termed the *Comparison* task, participants had to identify whether an Arabic (probe) digit was larger than either of two simultaneously presented comparator Arabic digits. This task and our speeded relative quantity task both involve the assessment of quantities given the visual presentation of digits. A key finding in the Tang et al. (2006) study was that different brain networks were identified for the Chinese and the English participants when they undertook the Comparison task. For the Chinese – but not the English participants – the authors identified a left-hemisphere network tracing a pathway from Wernicke’s area to the visual fusiform gyrus mediated by the premotor association (PMA) area. Tang et al. (2006) stated that the “… mental operation for transcoding the visual numerical codes to its semantic output codes may be different between NCS [Native Chinese Speakers] and NES [Native English Speakers]” (p. 10,777).

Tang et al. (2006) went further and speculated that the distinctive neural pathway in processing Arabic digits in the Chinese, but not the English, participants may arise due to the way in which Chinese children learn to read. They learn to read both Mandarin and Arabic digits in the context of learning the language’s character-based writing system. We agree that the distinct means by which children learn to read Chinese characters influences brain development in a very particular way. This difference may manifest in the conversion process that we identified here: the brain network described by Tang et al. (2006) may in fact reflect a neural region (e.g., the PMA) responsible for recovering a Mandarin representation associated with the Arabic digit. This, of course, is only speculation and should be explored further in future research.

Our data also allow us to predict particular patterns of performance in the same/different tasks reported by Cohen et al. (2013) if used with Chinese participants. Here we envisage tasks in which, on every trial, two numerals are presented, one above the other, and the participant must decide whether the two numerals represent the same quantity. In the same format conditions, the numerals are either a pair of Arabic digits (termed the *Arabic condition*) or a pair of Mandarin digits (termed the *Mandarin condition*). In these cases, the task can be completed simply be comparing the physical forms of the stimuli (i.e., quantity information is not necessary). Therefore, we expect that a significant predictor of performance in the Arabic condition will be PSA, and that a significant predictor of performance in the Mandarin condition will be PSM (after Cohen et al., 2013). In the *cross-format conditions*, one of the numerals is Arabic and the other is Mandarin. Because quantity information must be accessed to complete this condition, we expect PSM to be the primary predictor of performance. This follows from the current evidence for the Arabic to Mandarin translation process.

## Conclusions

In pursuing this line of research, we have again demonstrated the importance of how the surface forms of numbers critically determine how quantities are accessed. The approach initiated by Cohen (2009) demands that quantitative indices of physical similarity are derived in order that these indices can be used to model performance in various speeded numbers tasks. Here we have considered performance in a speeded relative quantity task and have assessed how Chinese participants deal with digits presented either in Arabic or Mandarin formats. In following Cohen (2009) we have, for the first time, derived indices of physical similarity for the key Chinese digits. We have shown that such indices provide statistically robust predictors of performance in the task.

Aside from these methodological advances, we have extended the reach of the Multiple Representation model of Cohen et al. (2013). Prior to the current work the model was able to provide an account of various number formats used by English language participants. Here we have shown how the model can also naturally account for the performance of Chinese participants. For these participants, who are acquainted with two different sets of numerals, it seems that both systems can act in a competitive way and that each has its own associated set of format-specific quantities.

### Open practices statement

The experiment was not preregistered. The data and analysis script are available at https://github.com/ccpluncw/ccpl_data_chineseNumber2019.git

## Appendix

It was of some additional interest to see how performance varied according to the order in which participants completed the two tasks. To this end the data were broken down according to whether the conditions were administered in the order Arabic then Mandarin (AM) or Mandarin then Arabic (MA).

### Summary of additional analyses

In considering the order in which the participants were exposed to the Arabic and Mandarin conditions the additional analyses show that order of testing did magnify one effect revealed in the overall analysis. In the overall analyses, the influence of PSA was only statistically reliable in the Arabic condition and not in the Mandarin condition. Furthermore it was opposite in direction to that predicted. We have interpreted this negative influence to reflect a suppressive effect that the Arabic number system exerts on the Mandarin system. The additional analyses show that the PSA effect was particularly sensitive to the order of testing because when the Arabic condition was tested second the PSA effect was nullified.

In the additional correlational analyses this particular order effect was reflected in the performance of the MA participants because in this group’s data for the Arabic condition the strength of the association between PSM and PSA was also nullified.

### Analyses in detail

#### Predictor fits in the Arabic condition

In the analysis of the data for the AM participants, for whom the Arabic condition was tested first, there was a statistically significant effect of the Welford function, (slope = 0.04), *F*(1, 214) = 117.76, *p* < .0001, and a statistically significant effect of the PSM, (slope = 0.13), *F*(1, 214) = 16.31, *p* = .0001. There was also an effect of the PSA, but in the opposite direction (slope = -0.02), *F*(1, 214) = 7.90, *p* < .01.

In the analysis of the data for the MA participants for who the Arabic condition was tested second, there was a statistically significant effect of the Welford function, (slope = 0.04), *F*(1, 221) = 197.54, *p* < .0001, and a statistically significant effect of the PSM, (slope = 0.01), *F*(1, 221) = 10.68, *p* < .01. There was however no effect of the PSA, (slope = -0.005), *F* < 1.0.

#### Predictor fits in the Mandarin condition

In the analysis of the data for the MA participants for who the Mandarin condition was tested first, there was a statistically significant effect of the Welford function, (slope = 0.06), *F*(1, 221) = 221.52, *p* < .0001, and a statistically significant effect of the PSM, (slope = 0.01), *F*(1, 221) = 11.60, *p* < .001. There was also an effect of the PSA, but in the opposite direction (slope = -0.01), *F*(1, 221) = 8.12, *p* < .01.

In the analysis of the data for the AM participants for who the Mandarin condition was tested second, there was a statistically significant effect of the Welford function, (slope = 0.05), *F*(1, 221) = 147.17, *p* < .0001, and a statistically significant effect of the PSM, (slope = 0.009), *F*(1, 221) = 9.09, *p* < .01. There was also an effect of the PSA, but in the opposite direction (slope = -0.014), *F*(1,221) = 8.10, *p* < .01.

#### Summary

To reiterate; the overall analyses showed that in both the Arabic and Mandarin conditions the same patterns of performance obtained. Namely, both the Welford function and PSM provided reliable and positive fits with the data in both conditions. In contrast, the PSA provided a robust fit for the data from the Mandarin condition and a marginal fit for the data in the Arabic condition. In both of these cases the fits were with respect to negative slopes.

When the data were broken down according to the order in which the sessions were tested the same overall patterns obtained for the fits associated with the Welford and the PSM in all cases. In general, negative slopes were found for the PSA, but the fit failed to reach statistical significance in the data for the MA participants in the Arabic condition (i.e., when this condition was tested second). This suggests the presence of a carry-over effect (see Poulton, 1974) such that if the participants were first entrained in processing Mandarin digits prior to being tested with Arabic digits, the influence of the Arabic number system was nullified following the switch to the Arabic condition.

### Correlational analyses as a function of order of testing.

#### Cross-condition comparisons

The correlation between the strength of influence in PSM across the Arabic and Mandarin conditions for the MA participants was statistically reliable, *r* = 0.44, *t*(30) = 2.70, *p* < .011. The same correlation for the AM participants was marginally significant, *r* = .41, *t*(29) = 2.39, *p* =.02 (cf. Fig. 3 top left-hand panel). These correlation coefficients were statistically equivalent, *z* = 0.17, *p* = .86.

The correlation between the strength of influence in PSA across the Arabic and Mandarin conditions for the MA participants was not statistically reliable, *r* = 0.36, *t*(30) = 2.10, *p* < .044, and neither was it statistically reliable for the AM participants *r* = 0.19, *t*(29) = 1.03, *p* = .31 (cf. Fig. 3 top right-hand panel).

#### Summary

The additional correlations fail to throw any further light on the overall patterns of performance reported previously. Generally speaking, the influence of PSM is more evident in the data than is the influence of PSA. The lack of a significant correlations for PSA across the conditions underscores the same null effect in the overall analyses (see Fig. 3, top right-hand panel).

#### Performance within the Mandarin condition

There were strong negative correlations between the PSM and PSA predictors regardless of whether the Mandarin condition was tested first or second, *r* = - . 52, *t*(30) = -3.30, *p* = .002, for the MA participants and, *r* = -.64, *t*(29) = -4.44, *p* < .001, for the AM participants. In this respect these findings underscore the negative relation show in the overall analyses (see Fig. 3, bottom left-hand panel). These correlations were also statistically equivalent, *z* = 0.69, *p* =.49.

#### Performance within the Arabic condition

The correlation coefficient between PSA and PSM in the data for the MA participants in the Arabic condition failed to reach statistical significance, *r* = -0.18, *t*(30) = 1.00, *p* > .05. In contrast, the correlation coefficient between PSA and PSM in the data for the AM participant was statistically reliable, *r* = -.68, *t*(29) = -5.02, *p* < .0001. The strength of association was greater in the data for the AM participants than the MA participants, *z* = 2.48, *p* = .01.

#### Summary

Perhaps the additional finding of most interest to emerge from these within condition correlations is the failure to find a significant association between PSA and PSM for the MA participants in the Arabic condition. This does accord with the overall model fits that take account of order reported in this Appendix and suggests that the influence of the Arabic number system is nullified when the Arabic condition is tested second.

## Footnotes

1 The fact that the measures of PSM do not show any systematic variation with the indices of the Welford function may be taken to argue that any effects of PSM are not confounded with and are, therefore, functionally independent from those of numerical distance.

2 In response to a reviewer’s comments, we also carried out extensive subsidiary analyses of the data which take account of the order of testing of the Arabic and Mandarin conditions and these are reported in the Appendix. These analyses do provide some additional insights into performance but, more generally, simply refine rather than alter the picture that emerges of performance from the overall analyses reported in the body of the paper.
